# Preferences and Safety Perceptions Regarding Food Packaging Among Portuguese Consumers

**DOI:** 10.3390/foods14234020

**Published:** 2025-11-24

**Authors:** Jennyfer Castillo, Leandro Oliveira, Cíntia Ferreira-Pêgo, João G. Costa, Ana S. Fernandes

**Affiliations:** 1 School of Health Sciences and Technologies, Universidade Lusófona, 1749-024 Lisboa, Portugal; 2 CBIOS, Universidade Lusófona’s Research Center for Biosciences & Health Technologies, 1749-024 Lisboa, Portugal

**Keywords:** food packaging, consumer perception, food safety, chemical migration, food contact materials, health risk

## Abstract

Packaging plays a crucial role in the food industry, primarily for product protection and safety. However, potentially harmful substances may migrate from packaging materials into food, raising concerns about human exposure and health risks. This study aimed to assess the preferences, awareness, knowledge, and safety perceptions regarding food packaging among a sample of Portuguese consumers. A cross-sectional study was conducted between April and May 2021, using an online questionnaire disseminated via social media networks. A total of 253 participants, aged between 18 and 56 years, were involved, with 124 studying or working in the health sciences field and 129 in other areas. It was found that preferences for bulk food options were shown by participants from the health sciences field, while those from other areas preferred plastic-packaged food. Additionally, a higher awareness of food safety concerns was demonstrated by participants from the health sciences field, while participants from other areas reported greater uncertainty. Moreover, participants from the health sciences field adopted more preventive practices to minimize exposure to packaging-related contaminants. This study highlights the importance of increasing consumer literacy on food contact materials and promoting safer practices to ensure safer food handling and packaging choices.

## 1. Introduction

Packaging plays a critical role in food production, ensuring product protection, maintaining quality [1,2], and safety standards throughout the supply chain [3]. Its main functions include extending shelf life, reducing food waste, facilitating transportation and storage, and providing nutritional information to consumers [4,5,6].

Food contact materials (FCMs) are materials intended to be in contact with food throughout the supply chain, including processing, packaging, distribution, storage, and consumption [6,7]. FCMs may contain intentionally added substances, which are deliberately used in manufacturing with a specific function, and non-intentionally added substances, which result from contamination, degradation, or by-products that may migrate into food [2,7].

Migration refers to the transfer of small amounts of substances from packaging into food matrices, potentially altering their properties and posing toxicological risks to consumers [8]. The European Food Safety Authority (EFSA) regulates this process through Specific Migration Limits (SML), which are based on toxicological assessments of substances used in packaging [9]. These limits are legally established under Regulation (EC) No. 1935/2004, ensuring the safety of FCMs [10]. Growing consumer awareness of health risks related to packaging materials has made migration a significant concern in recent years [1].

A wide range of materials, including plastics, glass, metals, and paper, are commonly used in the food industry due to their functional properties and chemical stability [7,11]. Among the materials used in the food industry are polymers such as Polyvinyl Chloride (PVC), Polyethylene Terephthalate (PET), and Polyethylene (PE), which, through appropriate processing and additives, form plastic materials. However, they can release harmful substances such as phthalates, which can disrupt endocrine and reproductive systems [12,13]. Bisphenol A (BPA) is a monomer widely used in the production of polycarbonate and epoxy resins, applied for instance as internal coatings of food cans. Chronic exposure to BPA has been associated with adverse health outcomes [14], and it remains one of the most studied materials in the context of FCMs. Chronic exposure to BPA may lead to adverse health effects, including neurological, reproductive, and cardiovascular toxicity, as well as an increased cancer risk [13,15]. The SML previously established for BPA in food (3 mg/kg) was reduced to 0.6 mg/kg in 2002 and further reduced to 0.05 mg/kg, based on updated scientific data from EFSA in 2015 [16,17]. Metal-based food contact materials can release BPA Diglycidyl Ether (BADGE) from can coatings, a compound associated with cytotoxic effects, neurotoxicity, and potential carcinogenicity [18,19]. Although considered more environmentally friendly, paper and cardboard materials can also transfer harmful substances such as phthalates, diisopropylnaphthalenes (DIPN), and benzophenone [19,20]. Benzophenone is classified as a possible human carcinogen [21], while DIPN toxicity has not been fully evaluated, warranting precautionary restrictions on their migration [22]. Glass packaging is generally considered chemically inert and safe; however, contamination by heavy metals such as lead and cadmium can occur, especially from glazes and printing inks [19]. These metals may be carcinogenic to humans, and strict regulatory limits have been established to prevent their migration into food. The migration of substances depends on several factors, including food chemical composition, storage temperature, duration of contact, and the surface area of the packaging material relative to the product [12,18].

To address increasing consumer concerns and improve food safety, alternative packaging methods, such as “smart packaging”, are being developed. These technologies use sensors and indicators to monitor the condition of products throughout the supply chain, detecting contamination, bacterial growth, and storage conditions [4,5,23]. Smart packaging, integrated with diagnostic systems via smartphones, has the potential to revolutionize food monitoring and tracking, offering real-time information on product quality and safety [24]. This can improve food safety by enabling early detection of contamination and deterioration, while also reducing food waste and increasing operational efficiency [25].

This study aimed to assess the preferences, knowledge, and safety perceptions of food packaging among Portuguese consumers. It further aimed to investigate whether packaging choices, purchasing habits, and safety perceptions are influenced by the area of educational background or professional activity.

## 2. Materials and Methods

### 2.1. Study Design and Population

This cross-sectional observational study utilized a questionnaire to assess Portuguese consumers’ preferences and perceptions regarding the safety of food packaging materials. The sample included individuals aged 18 to 56 years residing in Portugal. The questionnaire was available online from 28 April to 21 May 2021, yielding 253 responses. The study was approved by the Ethics Committee of the School of Health Sciences and Technologies of the Universidade Lusófona (EC.ECTS/P07.21).

### 2.2. Evaluation of Preferences and Perception of Food Packaging Safety

The questionnaire was created using Google^®^ Forms and disseminated through social media platforms such as LinkedIn^®^, X (Twitter)^®^, Facebook^®^, and WhatsApp^®^. It was structured into two main sections. The first part collected general characteristics of the population, such as age, gender, region of residence, and educational level, as well as questions regarding household composition and monthly income. Participants were also asked about their responsibility for grocery shopping, dietary habits, and food preferences. Additionally, participants were asked about their smoking habits and whether they worked or studied in the health sciences field.

The second part of the survey focused on participants’ practices and perceptions regarding food packaging safety. A 5-point Likert scale was used to assess the importance that participants attributed to various characteristics of food packaging, as well as to factors influencing the migration of chemicals from packaging into food. Participants were also asked about their usual packaging preferences for ten specific food items, providing insight into their choices and attitudes regarding packaging materials.

Regarding the safety of food packaging, participants were questioned about the potential migration of harmful substances into food and the associated health risks. Participants were asked to indicate the types of contamination they believed could occur based on the types of materials used in food packaging. Similarly, attempts were made to understand participants’ willingness to have more information regarding food packaging safety and whether they would be willing to pay more for food items contained in smart packaging.

### 2.3. Statistical Analysis

Statistical analysis was performed using Statistical Package for Social Sciences (IBM SPSS), version 26, for Windows (SPPS Inc., Chicago, IL, USA). Data were presented as percentages, % (absolute frequencies, n) for dichotomous variables, and mean (standard deviation, SD) for continuous variables. It was compared to the distribution of the selected characteristics between groups using Pearson’s Chi-squared test or Student *t*-test for categorical or continuous variables, respectively. All the analyses were performed according to the study or working area of the participants. All statistical tests were two-tailed, and the significance level was set at *p* < 0.05.

## 3. Results

This study included 253 participants, comprising 124 from the health sciences field and 129 from other fields. The mean age of the sample was 30.3 years (SD = 8.43). Participants’ professional or academic background categorized the general characteristics of the sample (Table 1). The sample was predominantly female (71.5%) and mainly resided in the Lisbon metropolitan area (58.9%). These characteristics were significantly more common among participants from the health sciences field (*p* < 0.001). Regarding educational level, 58.1% of participants from the health sciences field had a bachelor’s degree, compared to 55.8% of participants from other fields, who had lower academic qualifications (*p* < 0.001). At the family level, it was observed that participants from the health sciences field had households consisting of three to four people, while those from other fields had smaller households (*p* < 0.05). Regarding monthly income, most participants reported incomes between 1000 and 3000 euros, with the participants working or studying in the health sciences field reporting a higher average monthly family income (*p* < 0.001).

Concerning dietary habits, a difference in self-reported healthy eating behaviors was reported: 51.6% of participants from the health sciences field reported following a healthy diet, while 63.6% of participants from other fields indicated that they did not (*p* < 0.05). Regarding smoking habits, 74.3% of participants were non-smokers, being a higher percentage of smokers observed in the non-health-related field category (*p* > 0.001). No statistically significant differences were observed between the two groups, concerning age, involvement in grocery shopping, and preferences for food products.

Table 2 presents the distribution of consumers’ perceptions of various food packaging attributes, stratified by students/professionals from the health sciences field and individuals from non-health-related fields. Overall, most participants consider material safety (50.2%) and food preservation (54.2%) as “very important” aspects. In contrast, appearance was considered less relevant, with only 25.7% rating it as “very important”. Health sciences professionals valued material safety more (38.7% for “important” classification) compared to non-professionals (27.9% for the same classification level), while non-professionals assigned greater importance to environmental concerns (31.0% “very important” compared to 25.8% in health professionals). However, no statistically significant differences (*p* > 0.05) were observed between the groups for any of the listed packaging attributes.

The top 3 material preferences for the type of packaging during the purchase of different food items are shown in Table 3. Differences were most evident for fruits (*p* < 0.005) and tomatoes (*p* < 0.001), in which the preference for bulk packaging was observed in both groups. However, the preference was more pronounced among the participants from the health sciences field compared to those from other fields (68.1% vs. 49.2% for fruits, and 76.9% vs. 46.8% for tomatoes, respectively). Regarding leafy vegetables and carrots, participants from the health sciences field also significantly preferred these foods in bulk (57.5% for leafy vegetables and 67.8% for carrots), while participants from other fields significantly preferred plastic-packaged options (45.8% for leafy vegetables and 48.4% for carrots).

**Figure 1:**
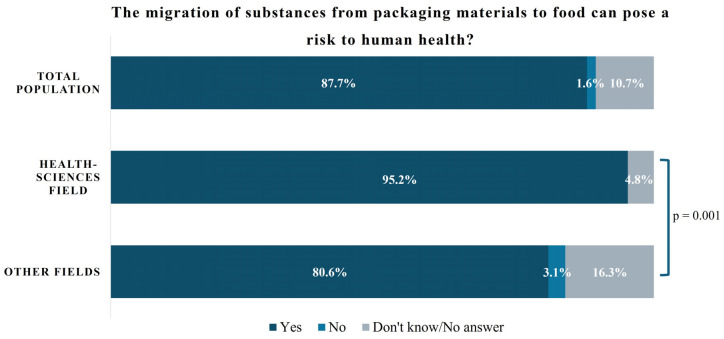
Knowledge about risk of substances migration into food, according to the study or the working area of the participants.

The knowledge about the health risks associated with the migration of chemicals from packaging materials can be observed in Figure 1. A significant statistical difference was observed between groups: the majority of respondents (87.7%) recognized migration as a potential health risk, with a notably higher awareness among professionals from the health sciences field (95.2%) compared to individuals from non-health-related fields (80.6%). Notably, disagreement with this risk was only reported among participants from non-health fields (3.1%).

The perception of the types of contamination that can occur in food based on the type of packaging material is presented in Table 4. Statistically significant differences were found regarding heavy metals contamination: 78.2% of participants from the health sciences field believed that such contamination could occur from tin/metal packaging, compared to 50.4% of participants from other fields. Interestingly, 6.5% of participants from the health sciences field, compared to 1.6% of participants from the non-health sciences field, believed that contamination could occur from glass packaging. Differences were also found regarding the risk of contamination with hormone-disrupting substances, especially among participants from the health sciences field (54.8%) compared to others (24.8%). For tin/metal cans, 41.9% of participants from the health sciences field identified a risk of endocrine disruption, compared to 24.8% of participants from other fields. Regarding contamination with carcinogenic substances, statistically significant differences were found for “plastic,” with 77.4% of participants from the health sciences field selecting this option, compared to 57.4% from other fields. A similar pattern was observed for tin/metal cans with 62.1% (health sciences field) vs. 38.0% (non-health sciences field) participants. Microbiological contamination was most attributed to paper/cardboard packaging (48.4% of participants from the health sciences field vs. 31.0% others), followed by plastic (48.4% vs. 29.5%) and glass (21.0% vs. 11.6%). In this study, some participants were unsure whether contamination could occur from packaging materials to food, with the most uncertainty related to endocrine-disrupting substances. Uncertainty was more prevalent among individuals from non-health-related fields. No significant statistical differences were found regarding contamination with pieces of packaging.

Table 5 shows the importance attributed to factors promoting migration from packaging materials. Significant differences between groups were found for temperature, contact time, type of material, and humidity, where the majority of the sample ranked these factors as “important” or “very important”. No significant differences were found between the importance attributed to packaging thickness, color, size, as well as the food properties, between those studying or working in the health sciences area and other fields.

**Figure 2:**
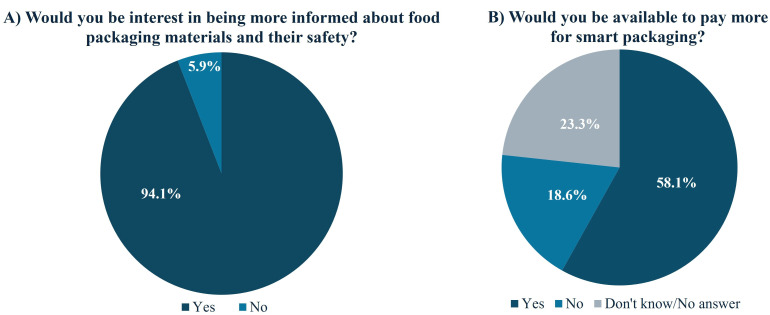
Need for information on food packaging safety (**A**) and willingness to pay for smart packaging (**B**).

The interest of the participants in receiving more information about packaging safety materials is presented in Figure 2A, with 94.1% of the participants expressing interest in being more informed about this topic. In addition, those working or studying in the health sciences field showed significantly more interest in receiving more information (97.6% vs. 90.7%, respectively) than those from other fields. Figure 2B shows the participants’ willingness to pay more for food packaged in smart packaging technologies, and no statistical differences were found between groups. However, the majority of the inquired individuals were willing to pay more for smart packaging.

An age-based sub-analysis showed significant differences in the perceived importance of the packaging “appearance” characteristics. Younger participants (18–27 years) were more likely to rate appearance as ‘very important’, whereas older participants (28–56 years) generally rated it as ‘important’. Similarly, the willingness to pay more for smart packaging also varied with age. Although the overall population expressed a general willingness to pay more, a subset of younger participants either indicated uncertainty or did not respond to this question.

## 4. Discussion

To the best of our knowledge, the present study is among the first to investigate consumer preferences and safety perceptions regarding food packaging materials in Portugal, revealing important findings about public awareness of FCMs. Participants from the health sciences field showed a higher preference for bulk fruits and vegetables, eventually suggesting greater concern with chemical migration and associated health risks. In contrast, participants not related to the health sciences field preferred packaged foods, particularly those packaged in plastic. This preference may be attributed to the convenience that plastic packaging offers, as it helps extend shelf life [26] and provides better protection of the product during transportation and storage [27].

FCMs must have strict safety criteria, ensuring that no harmful substances migrate into food under normal conditions of use [13]. However, plastic is not inert and can release potentially harmful substances into food (e.g., additives and degradation products), including phthalates, bisphenols, and residual monomers [28]. These can alter the organoleptic properties of food and present a health concern [26,28]. Thus, to reduce exposure to contaminants, a precautionary approach is recommended, which involves opting for fresh, unpackaged foods [13]. This is consistent with the findings of the current study, where the majority of participants recognized the potential health risks associated with migration, particularly among participants from the health sciences field. However, a small proportion of participants, mainly from non-health sciences fields, expressed uncertainty or disbelief in the relevance of packaging contamination.

This study also explored the perception of contamination from food packaging materials. Participants were more aware of heavy metals migration from glass and metal packaging. This is consistent with previous studies showing that canned foods, for example, can lead to the migration of heavy metals like tin into food [27,29]. Research by Ungureanu, Mustatea, and Popa [27] showed that although the levels of lead, cadmium, and chromium in packaging materials were below the allowed limits, the concentration of heavy metals in paper/cardboard packaging was higher compared to plastic, while glass packaging remained the most stable. Participants of this study did not specifically highlight plastic as a significant source of heavy metal contamination, despite growing evidence that certain pigments and stabilizers used in plastic manufacturing may contain metal residues [30].

Regarding endocrine disruptors, participants correctly identified plastic and metal packaging as potential sources of BPA and phthalates. These compounds are known to interfere with the endocrine system, potentially leading to adverse health effects, including reproductive and developmental issues, even at low doses [31,32]. BPA and phthalates can migrate from food packaging into food, with plastic packaging being the major source of exposure [13,33]. Recent findings suggest that the tolerable daily intake (TDI) for BPA may need to be revised due to evidence of immune and neurological effects [17]. This study’s findings align with the existing literature, as participants recognized the health risks of plastic packaging, but the potential of BPA migrating from paper/cardboard was not considered.

The risk of migration of carcinogenic chemicals from food packaging was also a concern for the participants of this study, which is consistent with the known migration of certain compounds, such as benzophenone, from paper/cardboard packaging [19,21]. The migration of these substances is well-documented, but the levels are generally below TDI [21]. However, to minimize long-term exposure, it is still recommended to limit consumption of foods stored in plastic or metal containers. The general population’s perception that certain FCMs, particularly plastics, could be a source of exposure to carcinogenic agents reflects the increasing consumer awareness. However, effective risk communication remains essential to contextualize low-dose exposure and possible cumulative effects.

Microbiological contamination was another concern highlighted by the participants in this study. Many respondents mentioned that contamination could occur from materials such as glass, paper/cardboard, and plastic. Pathogenic microorganisms are known to adhere to surfaces of food packaging materials, including stainless steel, plastic, and glass [18,21]. Paper/ cardboard packaging was considered the principal source of microbiological contamination, particularly due to fungi or bacteria that may migrate onto food [34]. However, previous studies have shown that paper/cardboard, when stored correctly, may reduce the potential for cross-contamination compared to plastic packaging, as the microbial viability decreases more rapidly [35].

The participants correctly identified key factors that promote the migration of harmful substances from packaging, including temperature, humidity, and contact time between the food and packaging [26,35]. This aligns with well-established findings in FCM toxicology, where migration kinetics increase under heat or prolonged contact. Surprisingly, less emphasis was placed on packaging thickness and food matrix characteristics, both of which significantly influence migration potential [36]. Research by Moura et al. [37] also echoed these concerns, noting that while consumers perceive stainless steel and glass as safer materials for cooking, plastic is still commonly used for food storage. Despite recognizing hazards, consumer behavior does not always align with risk perception. Bridging the gap between knowledge and behavior requires improved risk communication strategies. In Portugal, health literacy, defined as the ability to obtain, process, and understand health information, is often insufficient. A recent Portuguese study showed that nearly half of the population struggles with inadequate health literacy, limiting their ability to interpret food safety and packaging information or make informed choices [38]. This challenge is further reflected in studies on food labeling and nutritional literacy, where consumers recognize the importance of food labels but often fail to fully understand the information they contain [39,40]. This gap in understanding may explain the varying perceptions of food packaging safety observed in this study. Health and nutritional literacy remain key determinants of safe food handling and consumption. Those concepts are related to individuals’ ability to access and utilize nutritional information to maintain health [41,42]. Different studies have shown that individuals with higher levels of education, as experienced in the participants from the health sciences field, and specific dietary patterns tend to have better nutritional literacy. For example, Abreu et al. [43] reported that 84.1% of university students demonstrated adequate nutritional literacy, while Monteiro et al. [44] found that 65.2% of adults in Portugal exhibited good nutritional literacy, particularly among those with a healthy body mass index or those more familiar with nutrition. As demonstrated with this study, participants from the health sciences field showed a better understanding of this topic, which suggests the need to expand food packaging literacy efforts to the general public. Promoting food literacy, including knowledge of labeling, packaging materials, and safe storage practices, is essential to promote informed choices, leading to healthier outcomes and reducing the incidence of food-related health issues [38,41,45].

Beyond the dimensions assessed in this study, future research should incorporate consumer literacy and acceptance of smart/intelligent packaging, including perceived benefits (safety, freshness, waste reduction), barriers (cost, privacy, trust in sensors/indicators), and willingness to pay [46]. It is also important to examine post-consumer behaviours such as separation, recycling, reuse, and composting, perceptions regarding environmental labelling, and contamination of recycling streams. Moreover, exploring intergenerational transmission of knowledge and habits (e.g., from parents to children and caregivers to older adults) could clarify social influences on purchasing practices. The acceptance of edible packaging appears promising since consumer innovativeness strongly moderates adoption intentions [47]. Furthermore, consumer attitudes toward bioplastic food packaging highlight expectations for sustainability and a need for improved disposal literacy [48]. Finally, mapping the sources of information that shape consumer choices will be key to understanding how risk perception, consumer behaviour, and sustainability goals can be better aligned.

Despite the innovative character of this study, it presents some limitations that should be considered in the interpretation of results. Firstly, the sample may not accurately represent the broader adult population in Portugal due to the small size as well as the convenience sampling method, which could result in the overrepresentation or underrepresentation of certain demographic groups, thereby restricting the generalizability of the findings. Secondly, social desirability bias may have influenced participants’ responses, as they may provide answers, they believe are socially acceptable rather than reflecting their true opinions or behaviors. Finally, the cross-sectional design of the study does not allow the establishment of a cause–effect relationship. Future research could benefit from interventional studies that would provide greater comprehension into the dynamics between increased literacy on food packaging and subsequent behavioral changes. Educational campaigns, workshops, or informational materials focused on the risks and safety of FCMs could be effective in enhancing participants’ understanding of the potential health implications of food packaging [49]. Moreover, there is a need to develop and validate comprehensive tools to assess perceptions, attitudes, and behaviors related to food packaging and FCMs. Such tools could enable national and international comparisons of consumer attitudes and behaviors across different populations and settings and support evidence-based policy making in the field of consumer safety.

## 5. Conclusions

This study provides an important contribution to the understanding of consumer knowledge, perceptions, and practices related to FCMs and packaging materials safety in the Portuguese population. The main findings reveal that participants from the health sciences field, as informed consumers, demonstrated greater awareness regarding food packaging safety. Overall, most participants recognized migration from packing as a health risk. However, a minority, especially among those from non-health-related fields, did not perceive it as such. The importance of factors such as temperature, contact time, type of material, and humidity in promoting migration is widely acknowledged by most participants. However, there are notable gaps in knowledge concerning the risks associated with migration, particularly from commonly used plastic and paper-based materials. This work highlights the need for public initiatives to address scientific aspects of material safety and to bridge the translational gap between risk communication and consumer behavior. Promoting a culture of informed decision-making and shared responsibility between consumers, regulators, and the food industry is crucial to promoting public health in the presence of growing challenges associated with FCMs.

## Figures and Tables

**Table 1:** General characteristics according to the study or the working area of the participants.

	Total (n = 253)	Study/Working Field		*p*-Value ^a^
		Health-Sciences (n = 124)	Other Fields (n = 129)	
**Sex, % (n)**				
Female	71.5 (181)	84.7 (105)	58.9 (76)	**<0.001**
Male	28.1 (71)	15.3 (19)	40.3 (52)	
Other/No response	0.4 (1)	0.0 (0)	0.8 (1)	
**Age, years**	30.30 (8.43)	30.36 (8.63)	30.24 (8.26)	0.461
Age categories, % (n) *				0.784
18 to 27 years old	52.8 (133)	53.7 (66)	51.9 (67)	
28 to 56 years old	47.2 (119)	46.3 (57)	48.1 (62)	
**Residence, % (n)**				
North	3.6 (9)	2.4 (3)	4.7 (6)	**<0.001**
Center	19.0 (48)	16.1 (20)	21.7 (28)	
Lisbon Metropolitan Area	58.9 (149)	72.6 (90)	45.7 (59)	
Alentejo	1.6 (4)	2.4 (3)	0.8 (1)	
Algarve	13.4 (34)	5.6 (7)	20.9 (27)	
Azores Autonomous Region	0.8 (2)	0.0 (0)	1.6 (2)	
Madeira Autonomous Region	2.8 (7)	0.8 (1)	4.7 (6)	
**Education, % (n)**				
Basic/Secondary	39.9 (101)	23.4 (29)	55.8 (72)	**<0.001**
Bachelor’s degree	44.7 (113)	58.1 (72)	31.8 (41)	
Master’s/PhD	15.4 (39)	18.5 (23)	12.4 (16)	
Number of people in the household, % (n) *				
1–2	46.8 (118)	35.8 (44)	57.4 (74)	**0.001**
3–4	43.3 (109)	54.5 (67)	32.6 (42)	
≥5	9.9 (25)	9.8 (12)	10.1 (13)	
**Average monthly net family income, % (n)**				
<1000 €	22.9 (58)	11.3 (14)	34.1 (44)	**<0.001**
1000 to 3000 €	58.9 (149)	65.3 (81)	52.7 (68)	
>3000 €	11.1 (28)	16.9 (21)	5.4 (7)	
Don’t know/No response	7.1 (18)	6.5 (8)	7.8 (10)	
**Are you involved in purchasing groceries?, % (n)**				
Yes	91.3 (231)	93.5 (116)	89.1 (115)	0.214
No	8.7 (22)	6.5 (8)	10.9 (14)	
**Do you practice a healthy diet?, % (n)**				
Yes	43.9 (111)	51.6 (64)	36.4 (47)	**0.015**
No/Not always	56.1 (142)	48.4 (60)	63.6 (82)	
**Preference regarding food products, % (n)**				
Organic	6.7 (17)	8.1 (10)	5.4 (7)	0.466
Mixed	75.5 (191)	76.6 (95)	74.4 (96)	
Conventional	17.8 (45)	15.3 (19)	20.2 (26)	
**Smoking habits, % (n)**				
Smoker	25.7 (65)	15.3 (19)	35.7 (46)	**<0.001**
Non-smoker	74.3 (188)	84.7 (105)	64.3 (83)	

Data expressed in percentages (n) for categorical variables and mean (standard deviation) for continuous variables. ^a^ *p*-values for comparisons between groups were tested using Pearson’s Chi-squared test or Student *t*-test for categorical or continuous variables, respectively. * Missing values in age categories and number of household members variables (n = 1).

**Table 2:** Importance given to packaging characteristics according to the study or the working area of the participants.

	Total (n = 253)	Study/Working Field		*p*-Value ^a^
		Health-Sciences (n = 124)	Other Fields (n = 129)	
**Appearance, % (n)**				
Not important/Low importance	17.4 (44)	18.5 (23)	16.3 (21)	0.731
Moderately important	22.9 (58)	23.4 (29)	22.5 (29)	
Important	34.0 (86)	30.6 (38)	37.2 (48)	
Very important	25.7 (65)	27.4 (34)	24.0 (31)	
**Material safety, % (n)**				
Not important/Low importance	5.5 (14)	3.2 (4)	7.8 (10)	0.174
Moderately important	11.1 (28)	10.5 (13)	11.6 (15)	
Important	33.2 (84)	38.7 (48)	27.9 (36)	
Very important	50.2 (127)	47.6 (59)	52.7 (68)	
**Environmental concerns, % (n)**				
Not important/Low importance	9.5 (24)	6.5 (8)	12.4 (16)	0.204
Moderately important	19.8 (50)	20.2 (25)	19.4 (25)	
Important	42.3 (107)	47.6 (59)	37.2 (48)	
Very important	28.5 (72)	25.8 (32)	31.0 (40)	
**Convenience, % (n)**				
Not important/Low importance	12.3 (31)	9.7 (12)	14.7 (19)	0.481
Moderately important	19.8 (50)	22.6 (28)	17.1 (22)	
Important	39.1 (99)	37.9 (47)	40.3 (52)	
Very important	28.9 (73)	29.8 (37)	27.9 (36)	
**Food preservation, % (n)**				
Not important/Low importance	4.0 (10)	4.8 (6)	3.1 (4)	0.249
Moderately important	9.1 (23)	5.6 (7)	12.4 (16)	
Important	32.8 (83)	32.3 (40)	33.3 (43)	
Very important	54.2 (137)	57.3 (71)	51.2 (66)	
**Information provided, % (n)**				
Not important/Low importance	4.0 (10)	3.2 (4)	4.7 (6)	0.709
Moderately important	13.8 (35)	12.1 (15)	15.5 (20)	
Important	33.6 (85)	36.3 (45)	31.0 (40)	
Very important	48.6 (123)	48.4 (60)	48.8 (63)	
**Price, % (n)**				
Not important/Low importance	3.2 (8)	3.2 (4)	3.1 (4)	0.781
Moderately important	16.2 (41)	15.3 (19)	17.1 (22)	
Important	34.8 (88)	37.9 (47)	31.8 (41)	
Very important	45.8 (116)	43.5 (54)	48.1 (62)	

Data expressed in percentages (n). ^a^ *p*-values for comparisons between groups were tested using Pearson’s Chi-squared test.

**Table 3:** Top 3 material type preferences during food purchase according to the study or the working area of the participants.

	Total (n = 253)	Study/Working Field		*p*-Value ^a^
		Health-Sciences (n = 124)	Other Fields (n = 129)	
**Pulses, % (n)**				
Plastic	26.2 (50)	25.8 (24)	26.5 (26)	0.932
Can/Metal	49.2 (94)	48.4 (45)	50.0 (49)	
Glass	24.6 (47)	25.8 (24)	23.5 (23)	
**Fruits, % (n)**				
Plastic	33.9 (81)	28.6 (34)	39.2 (47)	**0.004**
Bulk	58.6 (140)	68.1 (81)	49.2 (59)	
No preference	7.5 (18)	3.4 (4)	11.7 (14)	
**Tomato, % (n)**				
Plastic	29.3 (63)	19.2 (20)	38.7 (43)	**<0.001**
Bulk	61.4 (132)	76.9 (80)	46.8 (52)	
No preference	9.3 (20)	3.8 (4)	14.4 (16)	
**Leafy vegetables, % (n)**				
Plastic	42.0 (100)	38.3 (46)	45.8 (54)	**0.006**
Bulk	49.2 (117)	57.5 (69)	40.7 (48)	
No preference	8.8 (21)	4.2 (5)	13.6 (16)	
**Carrots, % (n)**				
Plastic	38.8 (95)	28.9 (35)	48.4 (60)	**<0.001**
Bulk	53.5 (131)	67.8 (82)	39.5 (49)	
No preference	7.8 (19)	3.3 (4)	12.1 (15)	
**Mushrooms, % (n)**				
Plastic	46.7 (91)	48.5 (49)	44.7 (42)	0.535
Can/Metal	26.2 (51)	22.8 (23)	29.8 (28)	
Bulk	27.2 (53)	28.7 (29)	25.5 (24)	
**Flour, % (n)**				
Plastic	13.8 (33)	13.3 (16)	14.2 (17)	0.690
Paper/Cardboard	80.8 (194)	80.0 (96)	81.7 (98)	
No preference	5.4 (13)	6.7 (8)	4.2 (5)	
**Cod fish,% (n)**				
Plastic	47.6 (99)	44.0 (48)	51.5 (51)	0.548
Bulk	36.5 (76)	39.4 (43)	33.3 (33)	
Do not consume	15.9 (33)	16.5 (18)	15.2 (15)	
**Olive oil, % (n)**				
Plastic	26.4 (64)	27.1 (32)	25.8 (32)	0.946
Glass	69.8 (169)	69.5 (82)	70.2 (87)	
No preference	3.7 (9)	3.4 (4)	4.0 (5)	
**Juices, % (n)**				
Plastic	15.0 (31)	12.9 (13)	17.0 (18)	0.095
Tetrapak^®^/Paper/Cardboard	60.9 (126)	56.4 (57)	65.1 (69)	
Do not consume	24.2 (50)	30.7 (31)	17.9 (19)	

Data expressed in percentages (n). ^a^ *p*-values for comparisons between groups were tested using Pearson’s Chi-squared test. *p*-values for comparisons between groups were tested using Pearson’s Chi-squared test.

**Table 4:** Perception about the type of contamination that can occur in foods, considering the packaging material, according to the study or the working area of the participants.

	Total (n = 253)	Study/Working Field		*p*-Value ^a^
		Health-Sciences (n = 124)	Other Fields (n = 129)	
**Debris from packaging, % (n)**				
Glass	15.0 (38)	17.7 (22)	12.4 (16)	0.235
Paper/Cardboard	36.0 (91)	40.3 (50)	31.8 (41)	0.157
Can/Metal	17.8 (45)	20.2 (25)	15.5 (20)	0.333
Plastic	57.3 (145)	62.9 (78)	51.9 (67)	0.078
Wood	30.0 (76)	33.1 (41)	27.1 (35)	0.303
Does not occur	3.6 (9)	2.4 (3)	4.7 (6)	0.338
I don’t know	11.1 (28)	7.3 (9)	14.7 (19)	0.058
**Heavy metals, % (n)**				
Glass	4.0 (10)	6.5 (8)	1.6 (2)	**0.045**
Paper/Cardboard	4.3 (11)	3.2 (4)	5.4 (7)	0.391
Can/Metal	64.0 (162)	78.2 (97)	50.4 (65)	**<0.001**
Plastic	20.2 (51)	21.0 (26)	19.4 (25)	0.753
Wood	5.9 (15)	4.0 (5)	7.8 (10)	0.210
Does not occur	2.8 (7)	2.4 (3)	3.1 (4)	0.741
I don’t know	22.9 (58)	12.1 (15)	33.3 (43)	**<0.001**
**Hormone-disrupting substances, % (n)**				
Glass	2.8 (7)	1.6 (2)	3.9 (5)	0.273
Paper/Cardboard	5.5 (14)	5.6 (7)	5.4 (7)	0.939
Can/Metal	33.2 (84)	41.9 (52)	24.8 (32)	**0.004**
Plastic	39.5 (100)	54.8 (68)	24.8 (32)	**<0.001**
Wood	4.0 (10)	4.8 (6)	3.1 (4)	0.478
Does not occur	3.2 (8)	2.4 (3)	3.9 (5)	0.508
I don’t know	41.9 (106)	29.0 (36)	54.3 (70)	**<0.001**
**Carcinogenic substances, % (n)**				
Glass	2.4 (6)	2.4 (3)	2.3 (3)	0.961
Paper/Cardboard	14.2 (36)	13.7 (17)	14.7 (19)	0.817
Can/Metal	49.8 (126)	62.1 (77)	38.0 (49)	**<0.001**
Plastic	67.2 (170)	77.4 (96)	57.4 (74)	**0.001**
Wood	4.7 (12)	5.6 (7)	3.9 (5)	0.508
Does not occur	0.8 (2)	0.8 (1)	0.8 (1)	0.978
I don’t know	18.2 (46)	10.5 (13)	25.6 (33)	**0.002**
**Microbiological, % (n)**				
Glass	16.2 (41)	21.0 (26)	11.6 (15)	**0.044**
Paper/Cardboard	39.5 (100)	48.4 (60)	31.0 (40)	**0.005**
Can/Metal	31.6 (80)	35.5 (44)	27.9 (36)	0.195
Plastic	38.7 (98)	48.4 (60)	29.5 (38)	**0.002**
Wood	43.5 (110)	49.2 (61)	38.0 (49)	0.072
Does not occur	2.0 (5)	2.4 (3)	1.6 (2)	0.620
I don’t know	22.5 (57)	15.3 (19)	29.5 (38)	**0.007**

Data expressed in percentages (n). ^a^ *p*-values for comparisons between groups were tested using Pearson’s Chi-squared test.

**Table 5:** Importance attributed to factors promoting migration according to the study or the working area of the participants.

	Total (n = 253)	Study/Working Field		*p*-Value ^a^
		Health-Sciences (n = 124)	Other Fields (n = 129)	
**Temperature, % (n)**				
Not important/Less important	3.2 (8)	2.4 (3)	3.9 (5)	**0.044**
Moderately important	11.5 (29)	9.7 (12)	13.2 (17)	
Important/Very important	80.2 (203)	86.3 (107)	74.4 (96)	
I don’t know	5.1 (13)	1.6 (2)	8.5 (11)	
**Contact time, % (n)**				
Not important/Less important	5.1 (13)	3.2 (4)	7.0 (9)	**0.033**
Moderately important	14.6 (37)	14.5 (18)	14.7 (19)	
Important/Very important	73.9 (187)	79.8 (99)	68.2 (88)	
I don’t know	6.3 (16)	2.4 (3)	10.1 (13)	
**Thickness of the food contact layer, % (n)**				
Not important/Less important	8.3 (21)	5.6 (7)	10.9 (14)	0.060
Moderately important	19.8 (50)	24.2 (30)	15.5 (20)	
Important/Very important	56.5 (143)	58.9 (73)	54.3 (70)	
I don’t know	15.4 (39)	11.3 (14)	19.4 (25)	
**Material type, % (n)**				
Not important/Less important	2.8 (7)	1.6 (2)	3.9 (5)	**0.013**
Moderately important	9.9 (25)	11.3 (14)	8.5 (11)	
Important/Very important	79.8 (202)	84.7 (105)	75.2 (97)	
I don’t know	7.5 (19)	2.4 (3)	12.4 (16)	
**Humidity, % (n)**				
Not important/Less important	3.6 (9)	3.2 (4)	3.9 (5)	**0.006**
Moderately important	8.7 (22)	11.3 (14)	6.2 (8)	
Important/Very important	79.4 (201)	83.1 (103)	76.0 (98)	
I don’t know	8.3 (21)	2.4 (3)	14.0 (18)	
**Package color, % (n)**				
Not important/Less important	42.3 (107)	41.1 (51)	43.4 (56)	0.151
Moderately important	18.2 (46)	19.4 (24)	17.1 (22)	
Important/Very important	27.3 (69)	31.5 (39)	23.3 (30)	
I don’t know	12.3 (31)	8.1 (10)	16.3 (21)	
**Package size, % (n)**				
Not important/Less important	34.8 (88)	33.9 (42)	35.7 (46)	0.278
Moderately important	24.9 (63)	25.0 (31)	24.8 (32)	
Important/Very important	28.1 (71)	32.3 (40)	24.0 (31)	
I don’t know	12.3 (31)	8.9 (11)	15.5 (20)	
**Food properties, % (n)**				
Not important/Less important	4.3 (11)	4.8 (6)	3.9 (5)	0.716
Moderately important	9.9 (25)	10.5 (13)	9.3 (12)	
Important/Very important	71.1 (180)	72.6 (90)	69.8 (90)	
I don’t know	14.6 (37)	12.1 (15)	17.1 (22)	

Data expressed in percentages (n). ^a^ *p*-values for comparisons between groups were tested using Pearson’s Chi-squared test.

## Data Availability

The original contributions presented in this study are included in the article. Further inquiries can be directed to the corresponding author.
